# Supplementary material for: Single-cell RNA sequencing identifies shared differentiation paths of mouse thymic innate T cells
Source: Nat Commun. 2020 Aug 31;11:4367. doi: 10.1038/s41467-020-18155-8 (PMC7459300; doi:10.1038/s41467-020-18155-8)
Supplement: Supplementary file 1 — Supplementary Information [file 41467_2020_18155_MOESM1_ESM.pdf]

Supplementary information

Single-cell RNA sequencing identifies shared differentiation paths of mouse thymic innate T cells

Lee et al.

This file contains Supplementary Figures 1-20 and Supplementary Tables 1 and 2.

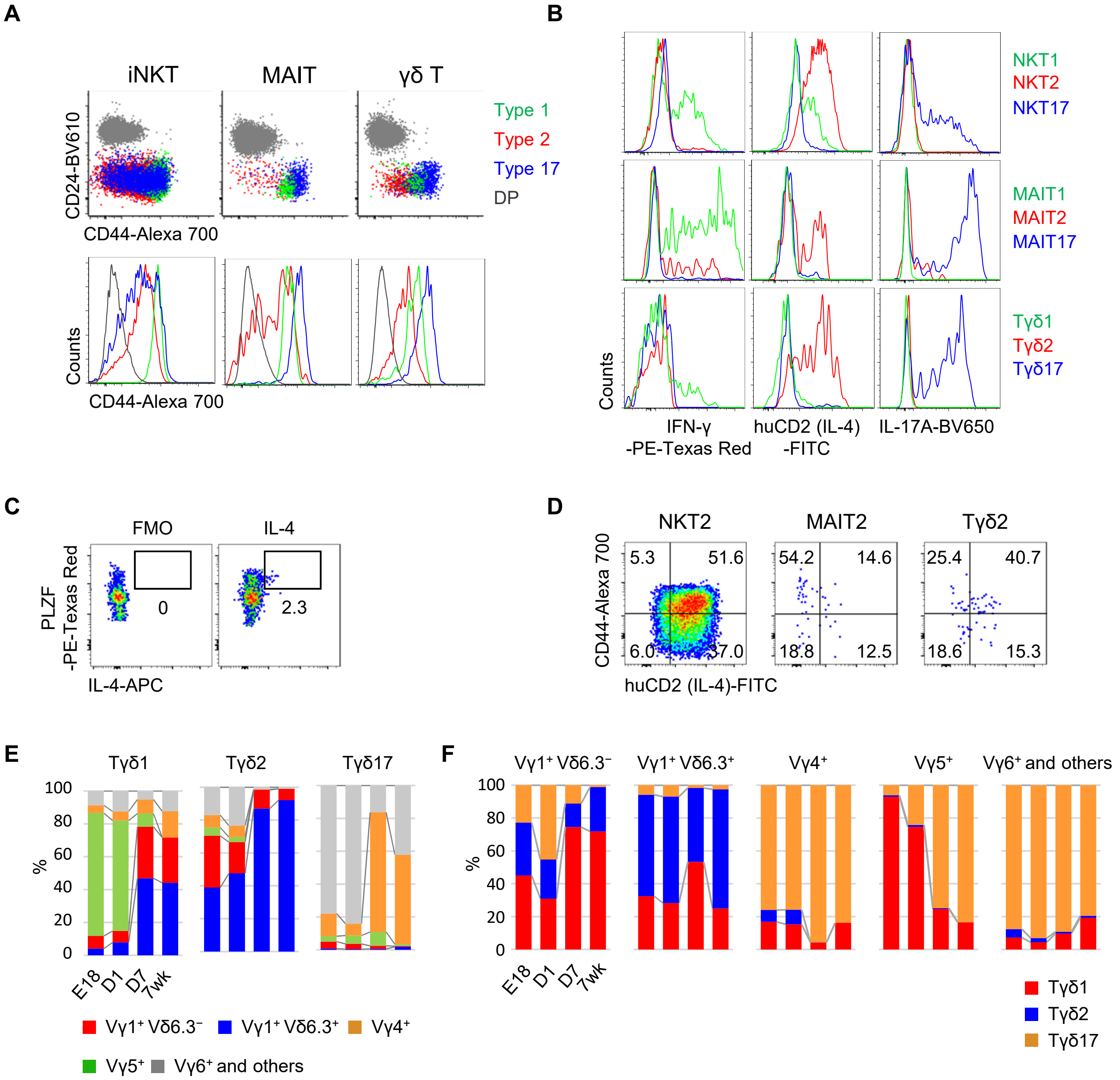

Supplementary Figure 1. Thymic development of innate T cells. (A) iNKT, MAIT and γδ T subsets gated in Figure 1A were analyzed for their expression of CD24 and CD44. Dot plots and histograms show representative results of at least five different experiments. (B and C) Thymocytes were stimulated with PMA/ ionomycin and indicated subsets gated as in Figure 1A were intracellularly stained with IFN-γ and IL-17A or surface stained with human CD2 (huCD2) in KN2 mice (B). CD24^{lo} PLZF^{+} MAIT cells gated as in Figure 1A were intracellularly stained with IL-4 (C) For MAIT cell cytokine analysis, 6-9 mice were pooled together. Experiments were repeated at least 3 times. (D) Dot plots show correlation between CD44 and huCD2 expressions in indicated cell types of KN2 mice in the thymus. (E and F) Tγδ1, Tγδ2 and Tγδ17 cells defined as shown in Figure 1A were analyzed for their usage of indicated TCRγδ chains at embryonic day 18 (E18, n=6), postnatal day 1 (D1, n=4), postnatal day 7 (D7, n=4), 7 week-old mice (7wk, n=3). Bar graphs show mean frequencies of indicated subset. Data were pooled from five independent experiments. Source data are provided as a Source Data file.

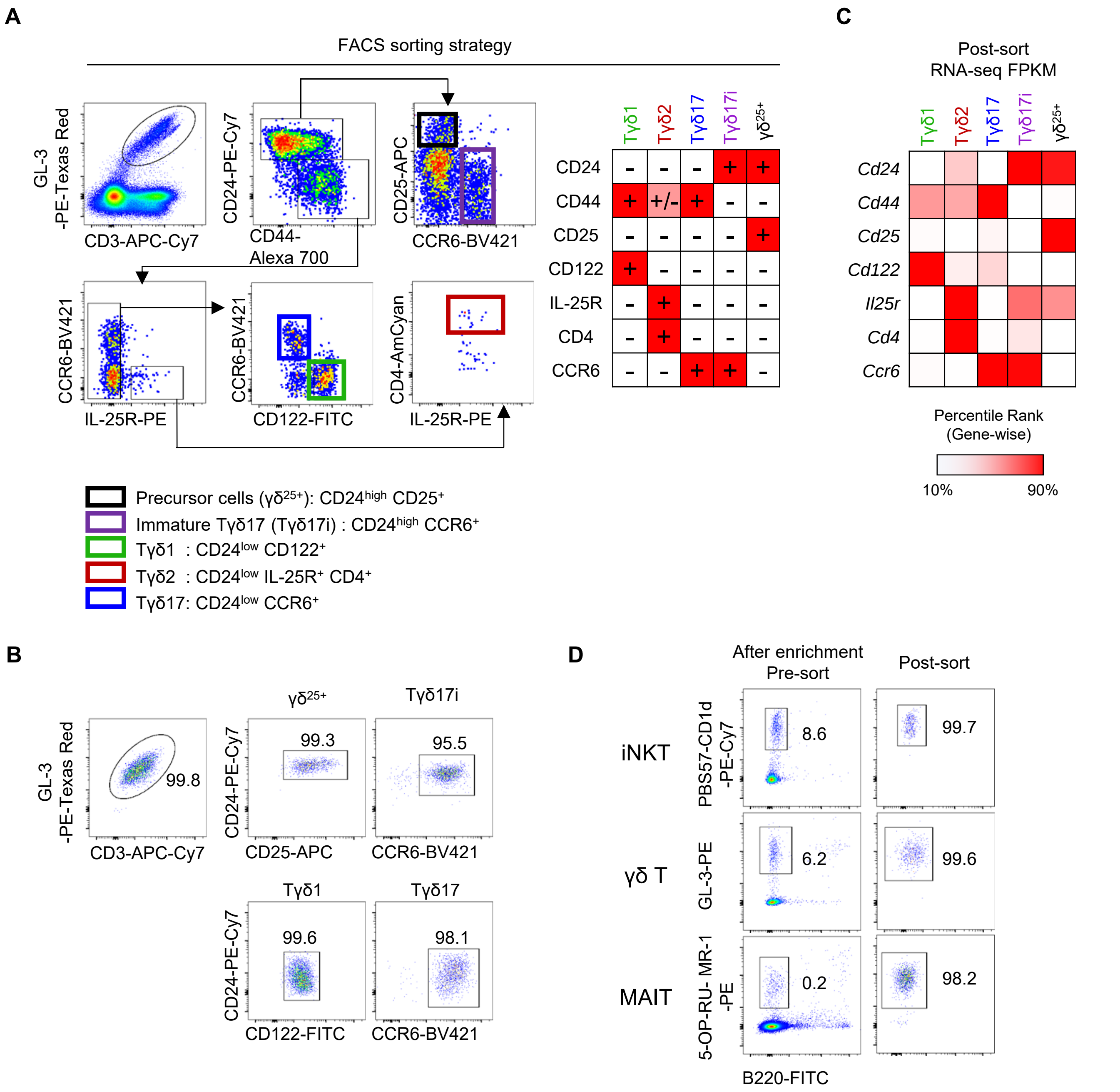

Supplementary Figure 2. Sorting strategy of γδ T cell subsets for bulk and scRNAseq. (A) γδ T cells were enriched from 5 day-old BALB/c thymocytes and stained with indicated markers. Five different subsets were sorted as shown in table and legend. (B) Dot plots show sorting purity of cells used for bulk-RNAseq. (C) Bulk RNA-seq FPKM values of markers used in the cell sorting were analyzed after sorting. (D) Single cell suspensions from thymi of BALB/c mice were enriched with total iNKT, γδ T, MAIT cells using MACS (left, pre-sort) and FACS sorted for single cell analysis (right, post-sort). Dot plots show their sorting purities. Numbers indicate frequency of cells in adjacent gates.

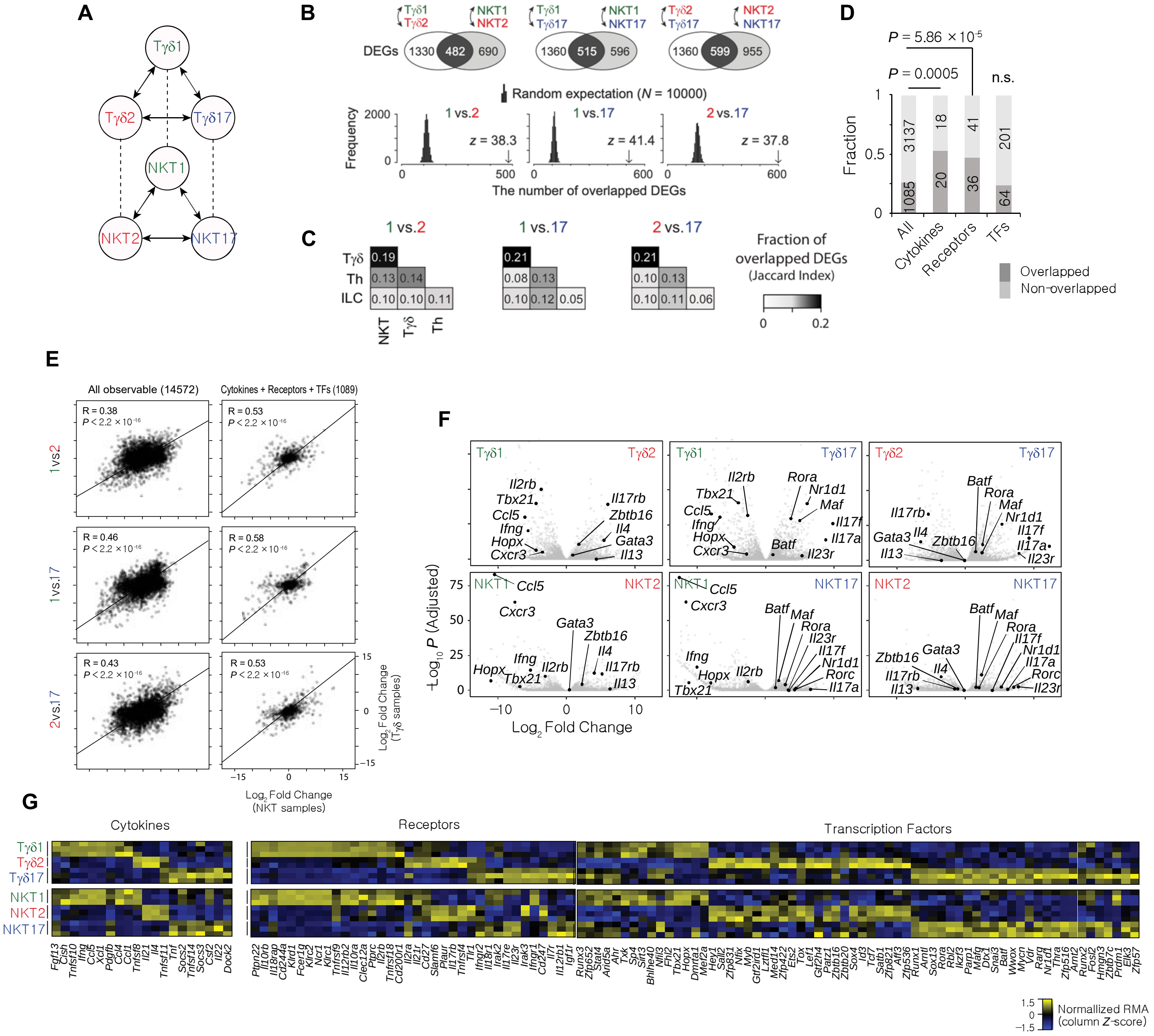

Supplementary Figure 3. iNKT and Tγδ subsets share transcriptional profiles. (A) Schematic figure depicts comparison strategy of iNKT and γδ T transcripts. Solid arrow represents pairwise comparisons of DEGs between subsets and dotted line presents numeric comparison of overlapped DEGs across γδ T and iNKT cells. (B) Venn-diagrams present the numeric comparison of overlapped DEGs across iNKT and γδ T cells. Bootstrapping analysis with shuffling of DEGs follows to show the statistical significance of the overlap. (C) Fractions of overlapped DEGs, calculated by Jaccard index, were compared among iNKT, γδ T, Th, ILC subsets. (D) Fractions of overlapped DEGs were compared among cytokines, receptors, transcription factors (TFs) and other genes. *P*-values were calculated with two-sided Fisher-exact test without multiple-test correction. (E) Scatter plots show gene expression ratios (log_2 of fold-change) between corresponded subsets across iNKT and γδ T cells. 'All observable' indicates mouse genes of which expression values are available in all samples. Cytokines + Receptors + TFs are union set of cytokine, receptor and transcription factor genes. (F) Volcano plots were generated after the pairwise comparison of indicated subsets. Genes that are known as DEGs in iNKT subsets were stressed and labeled. (G) Heat maps show expression patterns of cytokines, receptors and TFs mapped to overlapped differentially expressed genes (DEGs) in iNKT and γδ T cells.

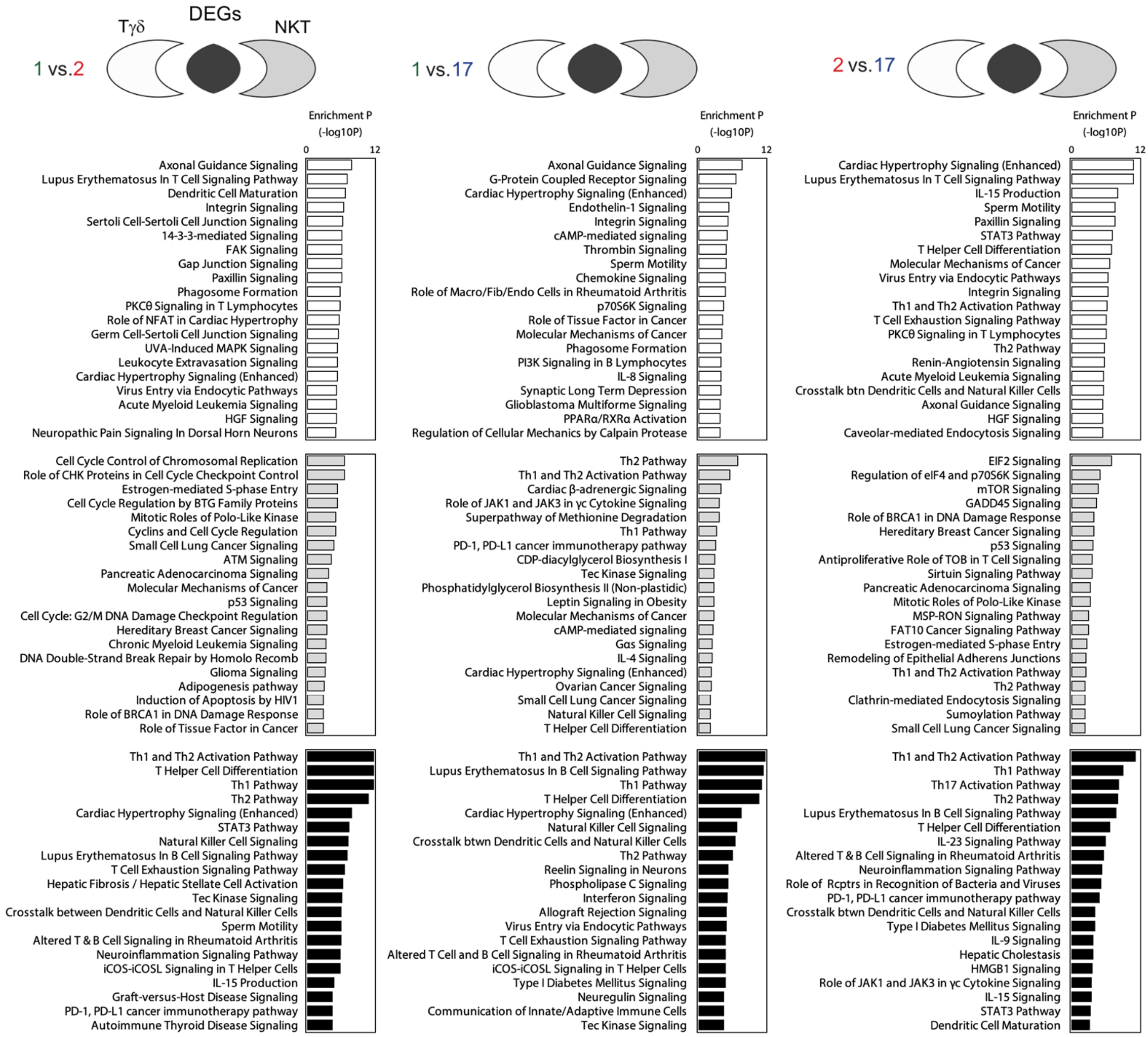

Supplementary Figure 4. Pathways that are distinct or common between γδ T and NKT linages. Functional enrichment studies were conducted in common, Tγδ–specific and NKT-specific DEGs between type 1 vs. type 2 (1 vs. 2), type 1 vs. type 17 (1 vs. 17) and type 2 and type 17 (2 vs. 17) subsets. Using IPA analysis, we listed top-20 significantly enriched canonical pathways (*P* < 0.05, one-tailed hypergeometric test with multiple-test correction).

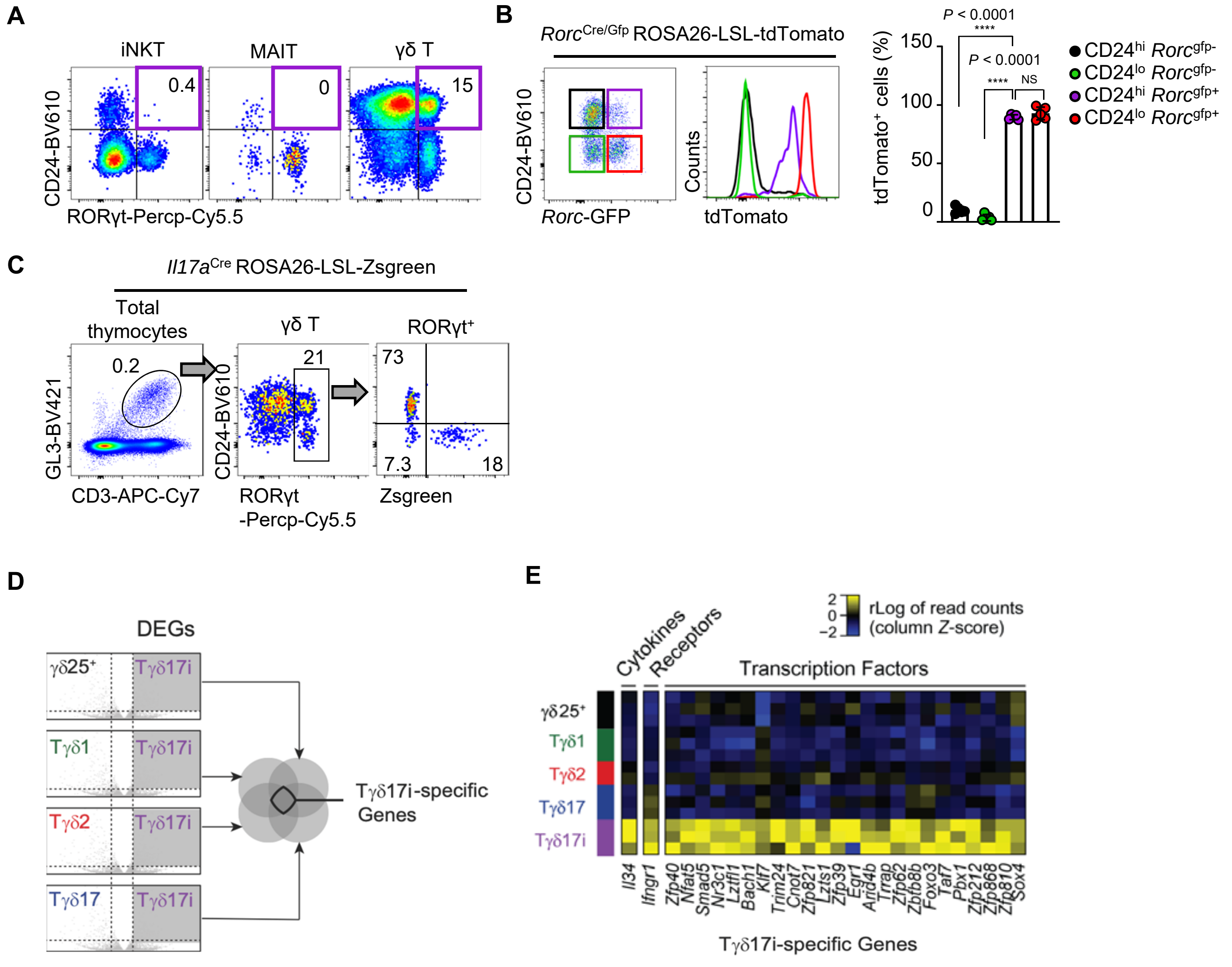

Supplementary Figure 5. Immature Tγδ17 cells express RORγt and have unique transcriptional signature. (A) Single cell suspensions of 7 week old BALB/c thymocytes were enriched with iNKT, MAIT and γδ T cells using MACS beads. Representative dot plots show CD24 and RORγt expression patterns of each cell type. Results are from at least five independent experiments. Numbers indicate frequencies of cells in adjacent gates. Data are presented as mean values ± SD. Unpaired two-tailed *t*-tests were used for data analysis. ****P<0.0001. NS, not significant. Source data are provided as a Source Data file. (B) Single cell suspensions of thymocytes from *Rorc*$^{Cre/Gfp}$ ROSA26-LSL-tdTomato mice were enriched with γδ T cells and analyzed for tdTomato expression in each subset. Representative dot plot and histogram are shown (left) and graph shows statistical analysis of frequencies of tdTomato expressing cells (right, n=5). Results from two independent sets of experiments are shown. (C) Representative dot plots show Zsgreen expression from CD24$^+$ and CD24$^-$ RORγt$^+$ thymic γδ T cells in *Il17a*^{Cre} ROSA26-LSL-Zsgreen mice. Representative data from three independent experiments are shown. (D and E) Tγδ17i cells were sorted as showed in supplementary figure 3 and analysed with other γδ T subsets. (D) Schematic figure shows how Tγδ17i-specific genes are generated from volcano plots. Tγδ17i-specific genes are from the intersection of DEGs overexpressed in Tγδ17i compared to other γδT subsets. (E) Heat map shows cytokine, receptors and transcription factors in Tγδ17i-specific genes.

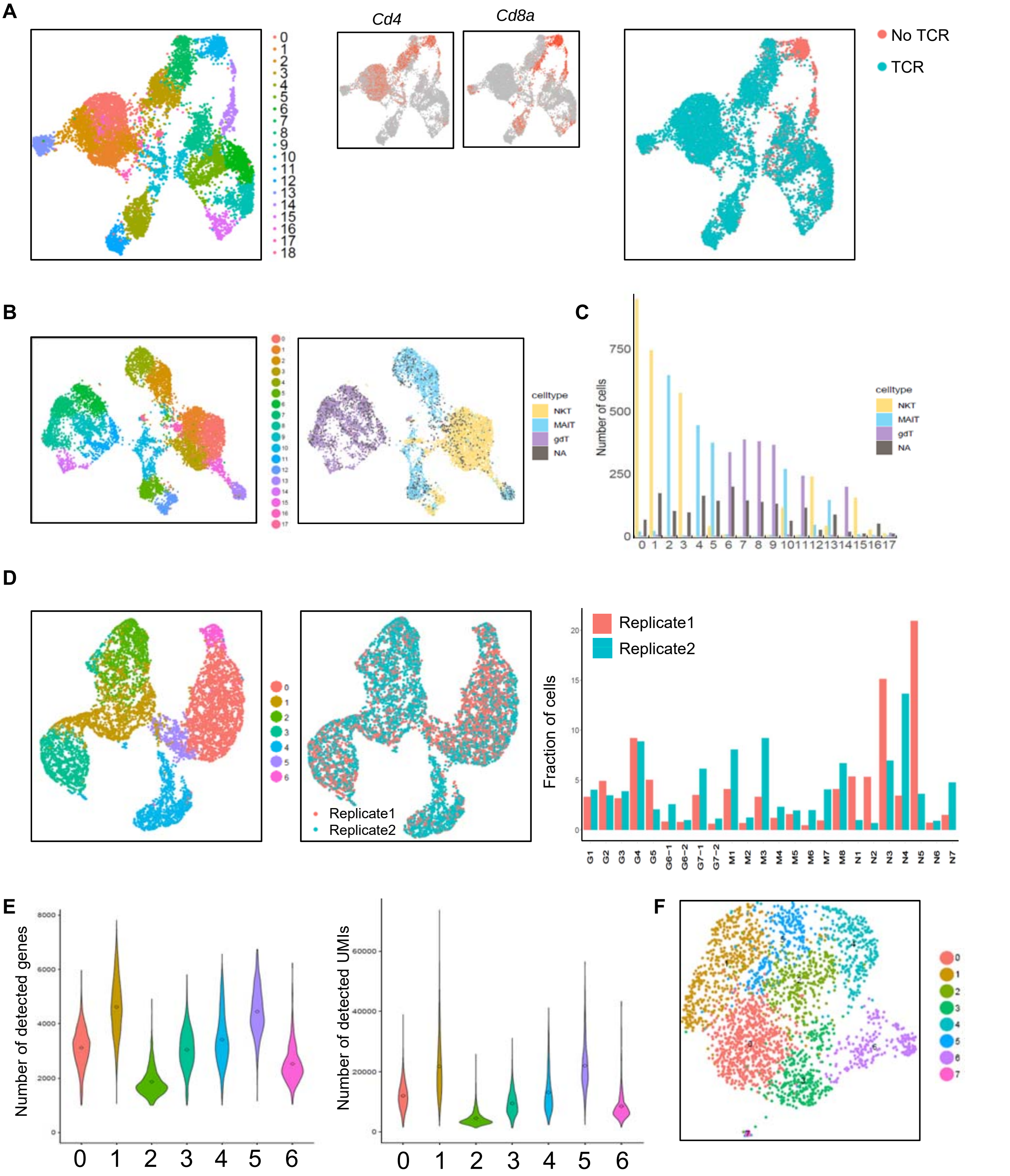

Supplementary Figure 6. Quality control for scRNA-seq. (A) UMAP plots show all cells before demultiplexing, which are colored by cell clusters (left), the expression levels of *Cd4* and *Cd8a* (middle) and the presence of detected TCRs (right). (B) UMAP plots of cells after excluding cluster 11 and 14 from (A), colored by reassigned cell clusters (left), demultiplexed cell types (right). (C) Bar plot shows the number of cells assigned to iNKT, MAIT, γδ T cells, or unassigned (NA) for each cell cluster. (D) UMAP plots show all assigned cells colored by cell clusters (left), replicates (center). Bat plot shows fractions of cells by replicates for each cell cluster of iNKT, MAIT, and γδ T cells (right). (E) Violin plots illustrate the number of detected genes (left) and UMIs (right) per cell for each cell cluster. (F) UMAP plot of γδ T cells showing an outlier cluster (cluster 7) which was removed from further analysis.

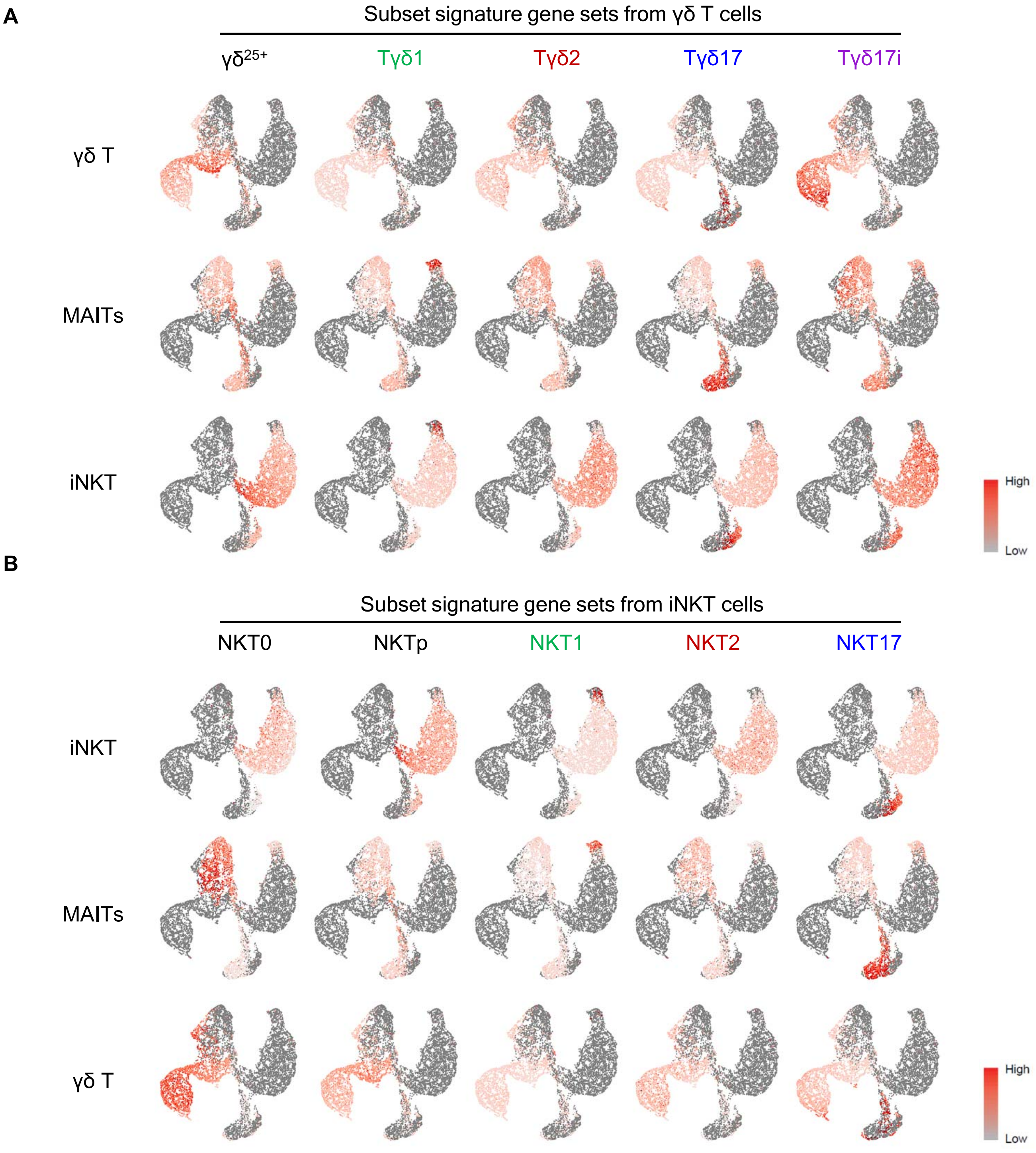

Supplementary Figure 7. Signature gene expression patterns of innate T cells. For each type of innate T cells, UMAP plots were colored by the signature scores defined by signature gene sets of functional subsets of γδ T cells (A) and iNKT cells (B).

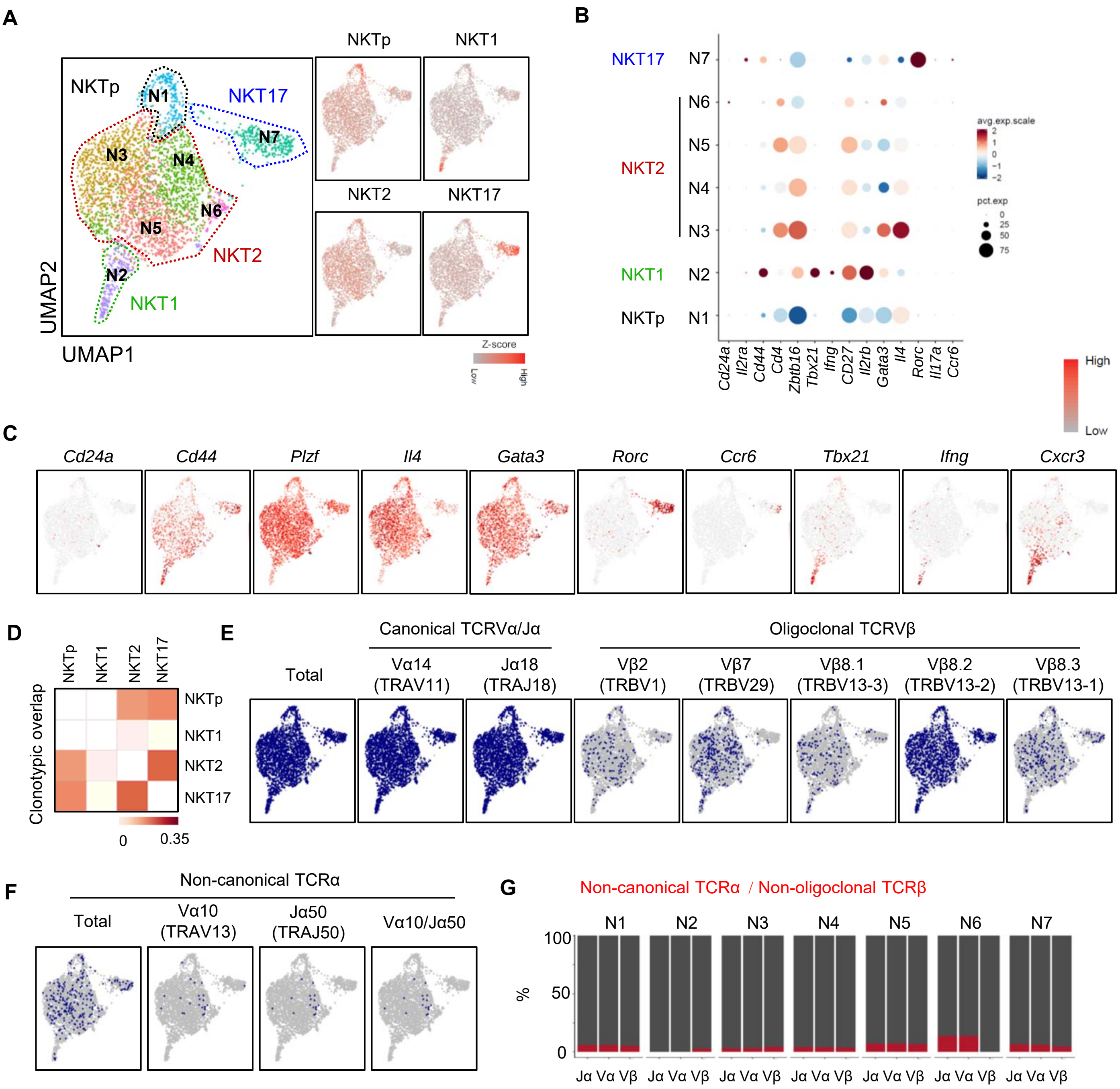

Supplementary Figure 8. Single cell analysis of iNKT cells. (A) UMAP plot is color-coded for each cell cluster (left, 1 panel) and signature scores of functional subsets of iNKT cells (right, 4 panels). (B) Dot plot shows the expression levels and frequencies of indicated genes in each cluster. The size and color of the dot represent the percentage of cells expressing the marker gene and the relative expression level (Z-score) within a cell cluster, respectively. (C) UMAP plots are colored by the expression level of indicated genes. (D) Heat map shows geometric mean of total frequencies of clonotypes that overlap between indicated subsets. (E-F) UMAP plots are colored by the presence of indiated TCR genes. (G) Bar graph shows percentage of cells expressing non-canonical TCRVα /Jα and non-oligoclonal TCRβ chains in each cluster.

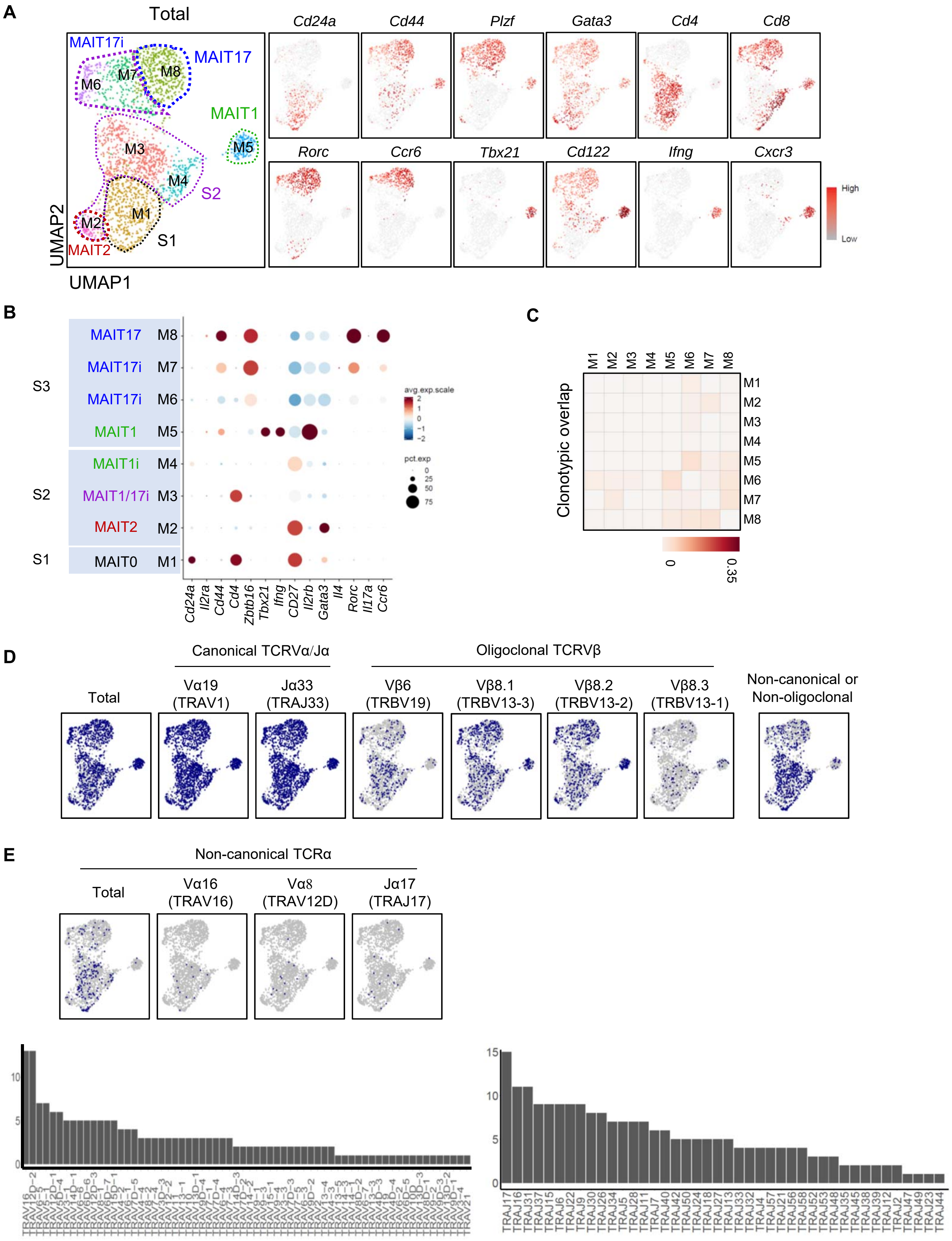
A
Total
MAIT17i
MAIT17
M6
M7
M8
MAIT1
M5
M3
M4
S2
M1
M2
MAIT2
S1
UMAP2
UMAP1
Cd24a
Cd44
Plzf
Gata3
Cd4
Cd8
Rorc
Ccr6
Tbx21
Cd122
Ifng
Cxcr3
High
Low
B
S3
S2
S1
MAIT17 M8
MAIT17i M7
MAIT17i M6
MAIT1 M5
MAIT1i M4
MAIT1/17i M3
MAIT2 M2
MAIT0 M1
avg.exp.scale
2
1
0
−1
−2
pct.exp
0
25
50
75
Cd24a
Il2ra
Cd44
Cd4
Zbtb16
Tbx21
Ifng
CD27
Il2rb
Gata3
Il4
Rorc
Il17a
Ccr6
C
M1
M2
M3
M4
M5
M6
M7
M8
Clonotypic overlap
0
0.35
D
Canonical TCRVα/Jα
Oligoclonal TCRVβ
Total
Vα19 (TRAV1)
Jα33 (TRAJ33)
Vβ6 (TRBV19)
Vβ8.1 (TRBV13-3)
Vβ8.2 (TRBV13-2)
Vβ8.3 (TRBV13-1)
Non-canonical or Non-oligoclonal
E
Non-canonical TCRα
Total
Vα16 (TRAV16)
Vα8 (TRAV12D)
Jα17 (TRAJ17)
0
5
10
15
TRAJ17
TRAJ16
TRAJ31
TRAJ37
TRAJ15
TRAJ6
TRAJ22
TRAJ9
TRAJ30
TRAJ26
TRAJ34
TRAJ5
TRAJ28
TRAJ11
TRAJ7
TRAJ40
TRAJ42
TRAJ50
TRAJ24
TRAJ18
TRAJ27
TRAJ13
TRAJ33
TRAJ32
TRAJ4
TRAJ57
TRAJ21
TRAJ56
TRAJ58
TRAJ52
TRAJ53
TRAJ48
TRAJ35
TRAJ45
TRAJ38
TRAJ39
TRAJ12
TRAJ2
TRAJ47
TRAJ49
TRAJ23
TRAJ44

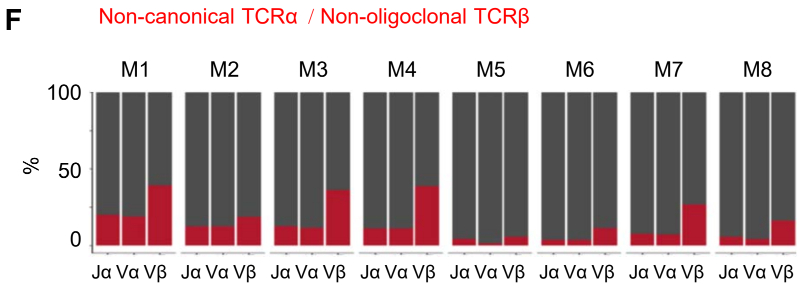

Supplementary Figure 9. Single cell analysis of MAIT cells. (A) UMAP plot is color-coded for each cell cluster (left, 1 panel). Expression levels of indicated genes were colored in UMAP (right, 12 panels). (B) Dot plot shows the expression levels and frequencies of indicated genes in each cluster. The size and color of the dot represent the percentage of cells expressing the marker gene and the relative expression level (Z-score) within a cell cluster, respectively. (C) Heat map shows geometric mean of total frequencies of clonotypes that overlap between indicated subsets. (D) UMAP plots are colored by the presence of indicated TCR genes. (E) UMAPs show the presence of indicated TCRs (top) and graphs show the distribution of frequently used non-canonical TCR Vα and Jα genes (bottom). (F) Bar graph shows percentage of cells expressing non-canonical TCRVα /Jα and non-oligoclonal TCRβ chains in each cluster.

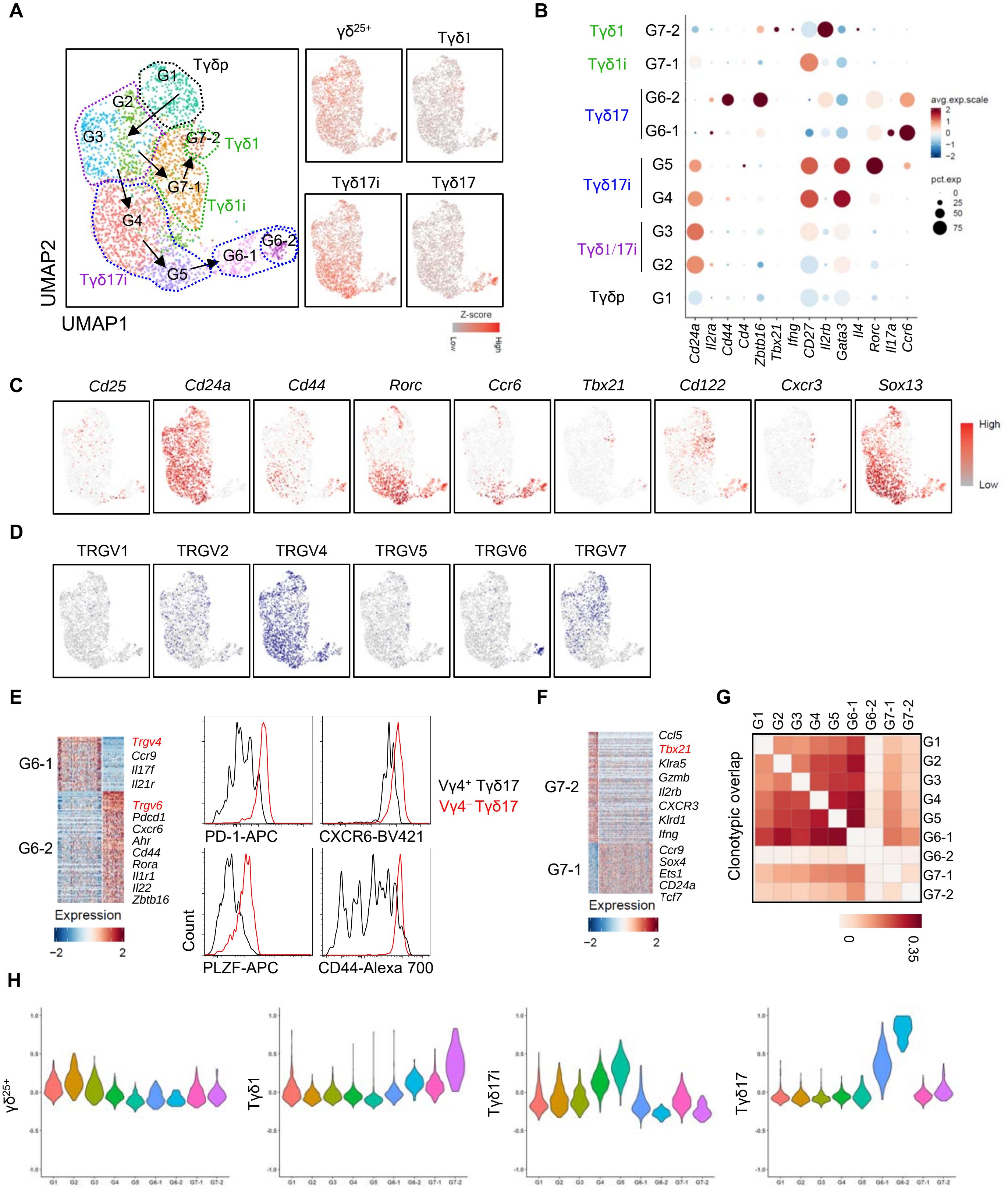

A
B
C
D
E
F
G
H
Tγδp
Tγδ1
Tγδ1i
Tγδ17
Tγδ17i
Tγδ1/17i
γδ25+
UMAP1
UMAP2
Z-score
avg.exp.scale
pct.exp
Cd25
Cd24a
Cd44
Rorc
Ccr6
Tbx21
Cd122
Cxcr3
Sox13
TRGV1
TRGV2
TRGV4
TRGV5
TRGV6
TRGV7
Vγ4+ Tγδ17
Vγ4− Tγδ17
PD-1-APC
CXCR6-BV421
PLZF-APC
CD44-Alexa 700
Count
Expression
Clonotypic overlap

Supplementary Figure 10. Single cell analysis of γδ T cells. (A) UMAP plot is color-coded for each cell cluster (left, 1 panel) and signature scores of functional subsets of γδ T cells (right, 4 panels). (B) Dot plot shows the expression levels and frequencies of indicated genes in each cluster. The size and color of the dot represent the percentage of cells expressing the marker gene and the relative expression level (Z-score) within a cell cluster, respectively. (C) UMAP plots are colored by the expression level of indicated genes. (D) UMAP plots colored by the presence of indicated TRGV genes. (E) Heat map shows the relative expression of differentially expressed genes between G6-1 and G6-2 (left). The histograms show the representative expression profiles of indicated markers on the $V\gamma4^{+}$ or $V\gamma4^{-}$ Tγδ17 cells of BALB/c thymocytes from three independent experiments (right). (F) Heat map shows the relative expression of differentially expressed genes between G7-1 and G7-2. (G) Heat map shows geometric mean of total frequencies of clonotypes that overlap between indicated subsets. (H) Violin plots showing $\gamma\delta^{25+}$, Tγδ1, Tγδ17i and Tγδ17 signature scores for each cell cluster of γδ T cells.

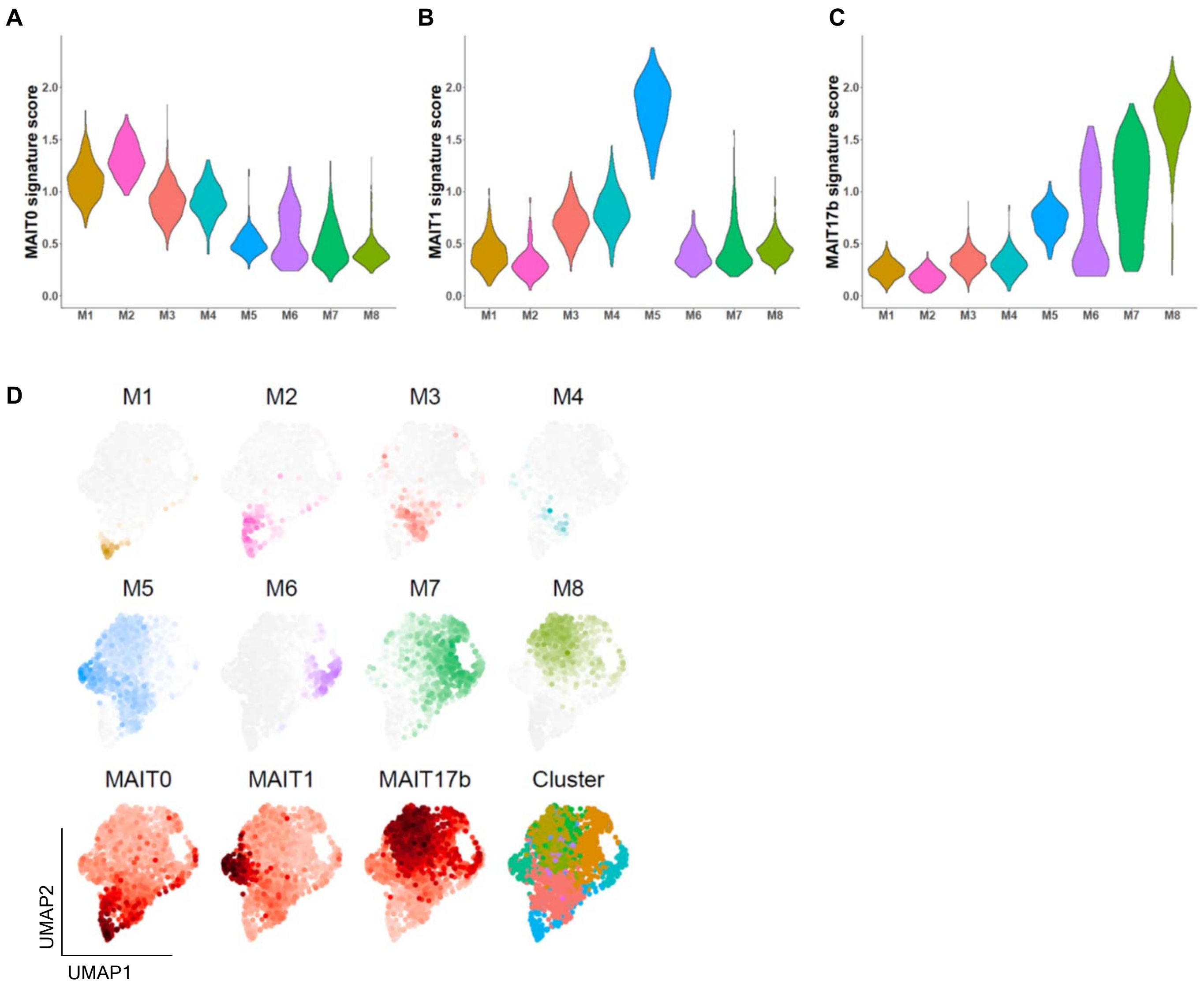

Supplementary Figure 11. Sex difference of MAITs. (A-C) Violin plots showing MAIT0 (A), MAIT1 (B), and MAIT17b (C) signature scores defined by Legoux et al. (Ref #13) for each cell cluster of MAITs. (D) UMAP plots show cells from the first replicate of wild-type scRNA-seq data of Legoux et al. (a mixture of male and female MAITs), which are colored by signature scores defined by our cell cluster of MAITs (M1 ~ M8), signature gene sets (MAIT0, MAIT1, MAIT17b) of Legoux et al (MAIT0, MAIT1 and MAIT17b), and new cell clusters ('Cluster'). Low-quality cells with greater than 4% of UMIs assigned to mitochondrial genes were removed. HVGs were identified using the decomposeVar function of the scran package with FDR ≤ 0.05 and biological variability > 0.05. Clusters were found using the FindClusters function of the Seurat package on the first 50 PCs of HVGs with resolution = 2.0.

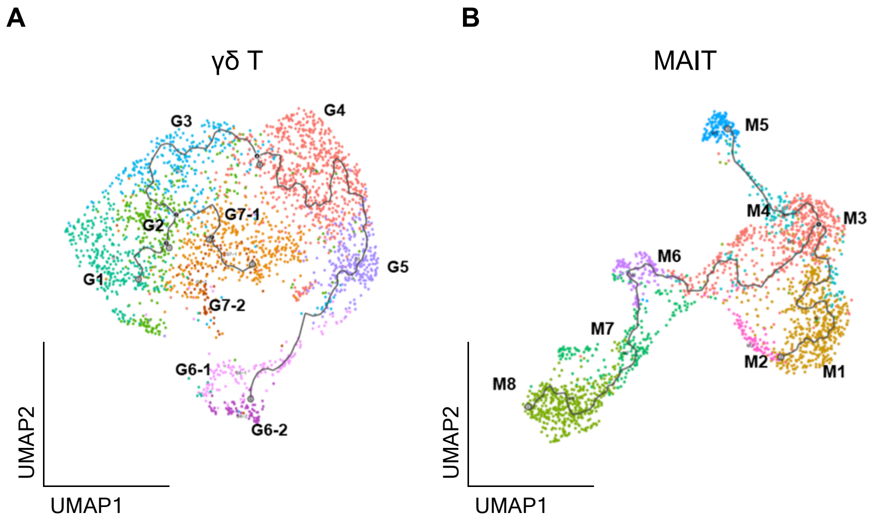

Supplementary Figure 12. Validation of inferred differentiation trajectories of MAIT and γδ T cells. UMAP plots showing differentiation trajectories of γδ T (A) and MAIT cells (B) by Monocle3. Cells were colored by clusters.

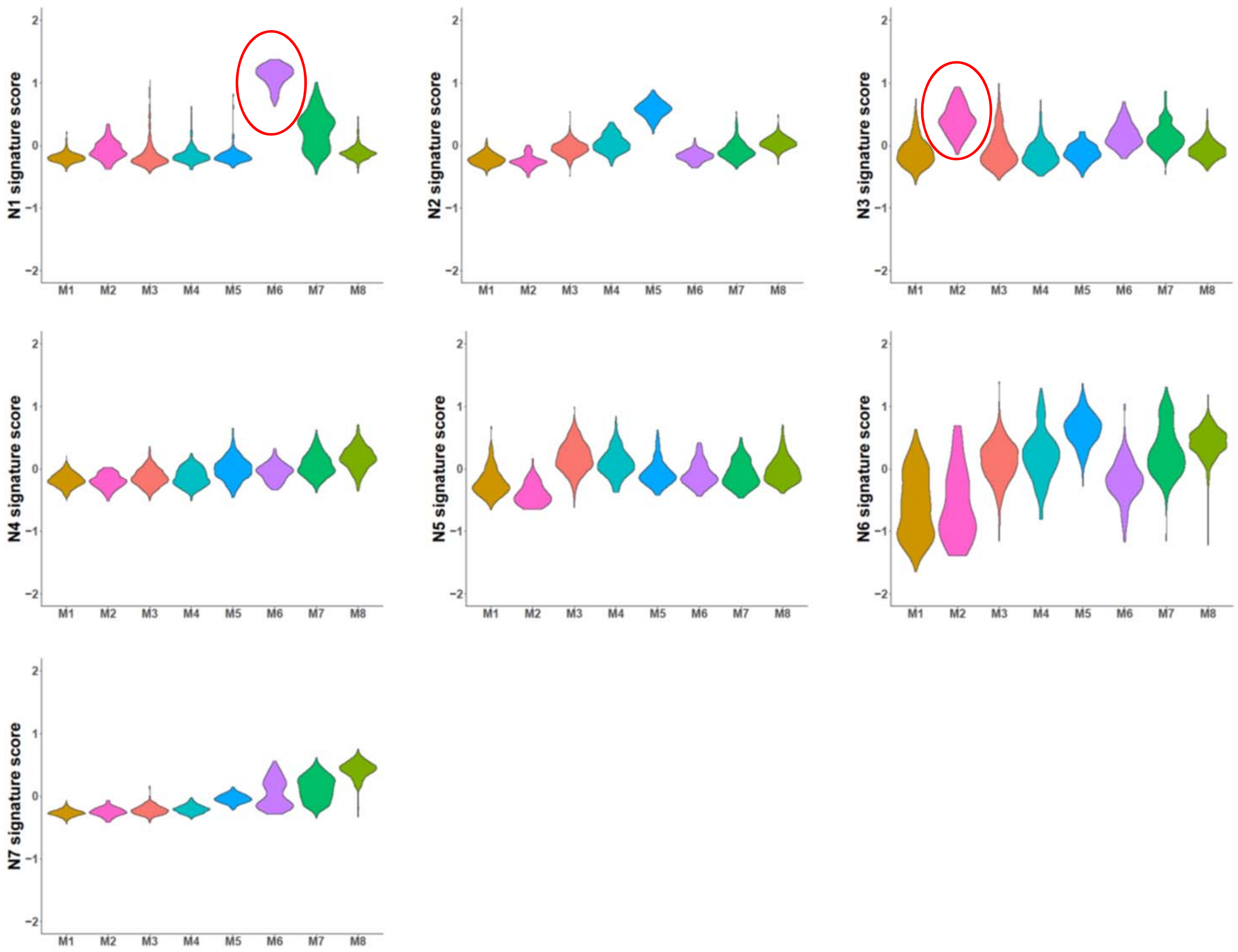

Supplementary Figure 13. Correlation between NKT and MAIT subsets. Graphs show signature scores of each NKT cluster in each MAIT cluster.

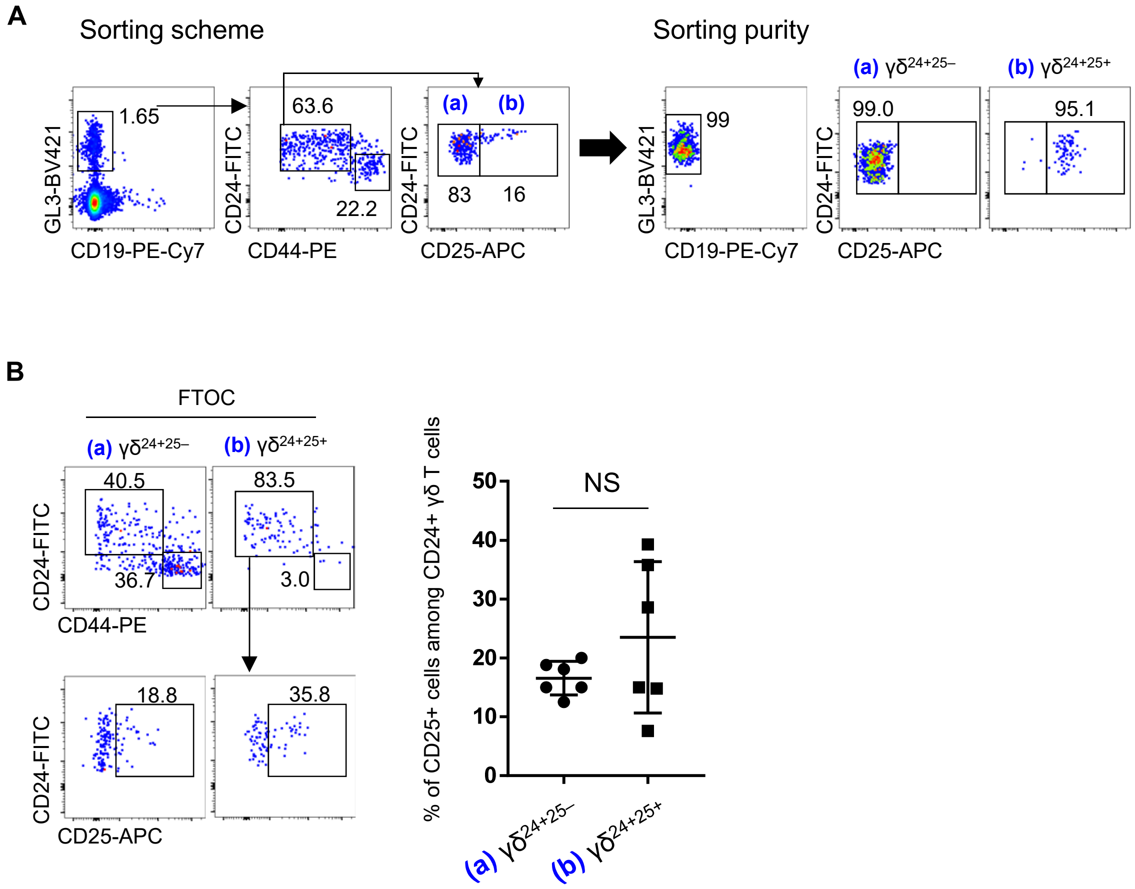

Supplementary Figure 14. $\gamma\delta^{24+25-}$ cells give rise to $\gamma\delta^{24+25+}$ cells in FTOC experiment. $\gamma\delta^{24+25-}$ and $\gamma\delta^{24+25+}$ cells were sorted from neonatal thymi of B6 mice and cultured in FTOC for 5 days. (A) Dot plots show sorting scheme and representative sorting purities. (B) Dot plots show the frequency of $CD24^+CD25^+$ cells among $CD24^+$ γδ T cells after FTOC experiments in each group (left). Graph shows the frequencies of $CD24^+CD25^+$ cells generated from $\gamma\delta^{24+25-}$ (a, n=6) and $\gamma\delta^{24+25+}$ (b, n=6). Results were pooled from four independent experiments. Numbers indicate frequencies of cells in adjacent gates. Data are presented as mean values ± SD. Unpaired two-tailed *t*-tests were used for data analysis. *P<0.05. NS, not significant. Source data are provided as a Source Data file.

Cumulative Distribution Function (clonotype > 1)

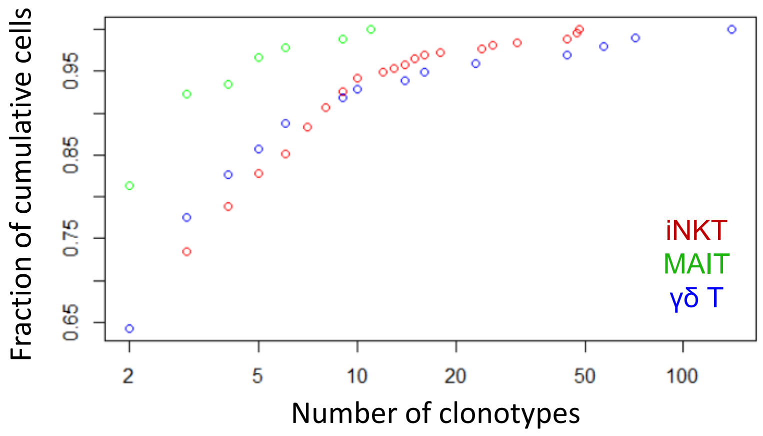

KS test	NKT-γδ T	γδ T-MAIT	NKT-MAIT
P value (Bonferroni)	1.000	0.388	0.001

Supplementary Figure 15. MAIT cells are more oligoclonal than iNKT or γδ T cells. Graph shows fraction of cumulative cells repeated more than once from Figure 4C. Table shows statistical analysis (two-sided Kolmogorov-Smirnov tests).

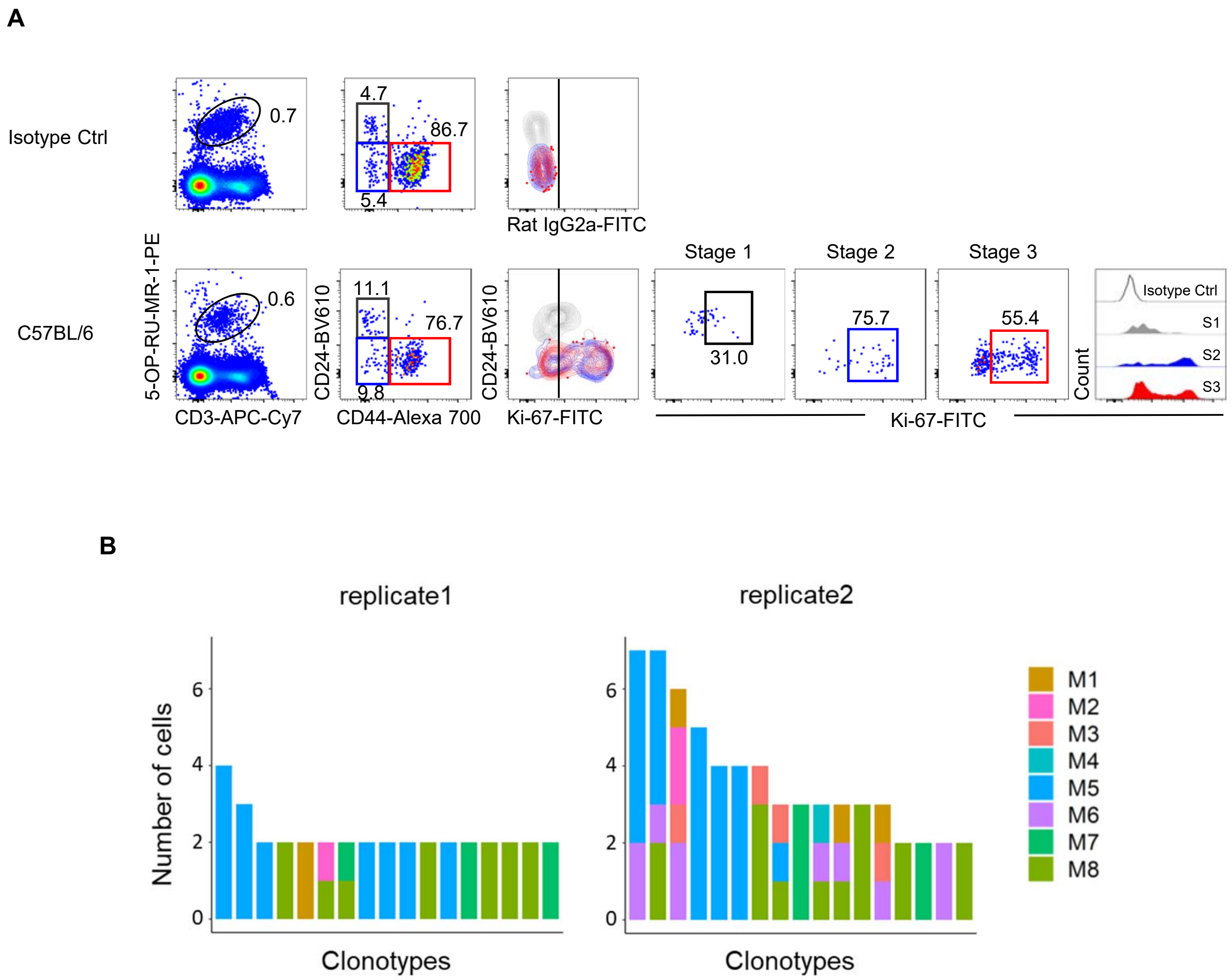

Supplementary Figure 16. Ki-67 expression and clonal repeat of MAIT cells. (A) Total thymocytes were enriched with MAITs and dot plots and histogram shows for their expression of Ki-67 in each stage. Representative results are from four independent experiments. (B) Bar plots show the ordered number of cells for each clonotype repeated 2 or more in each replicate of MAIT cells colored by UMAP regions. Each bar represents an individual clonotype.

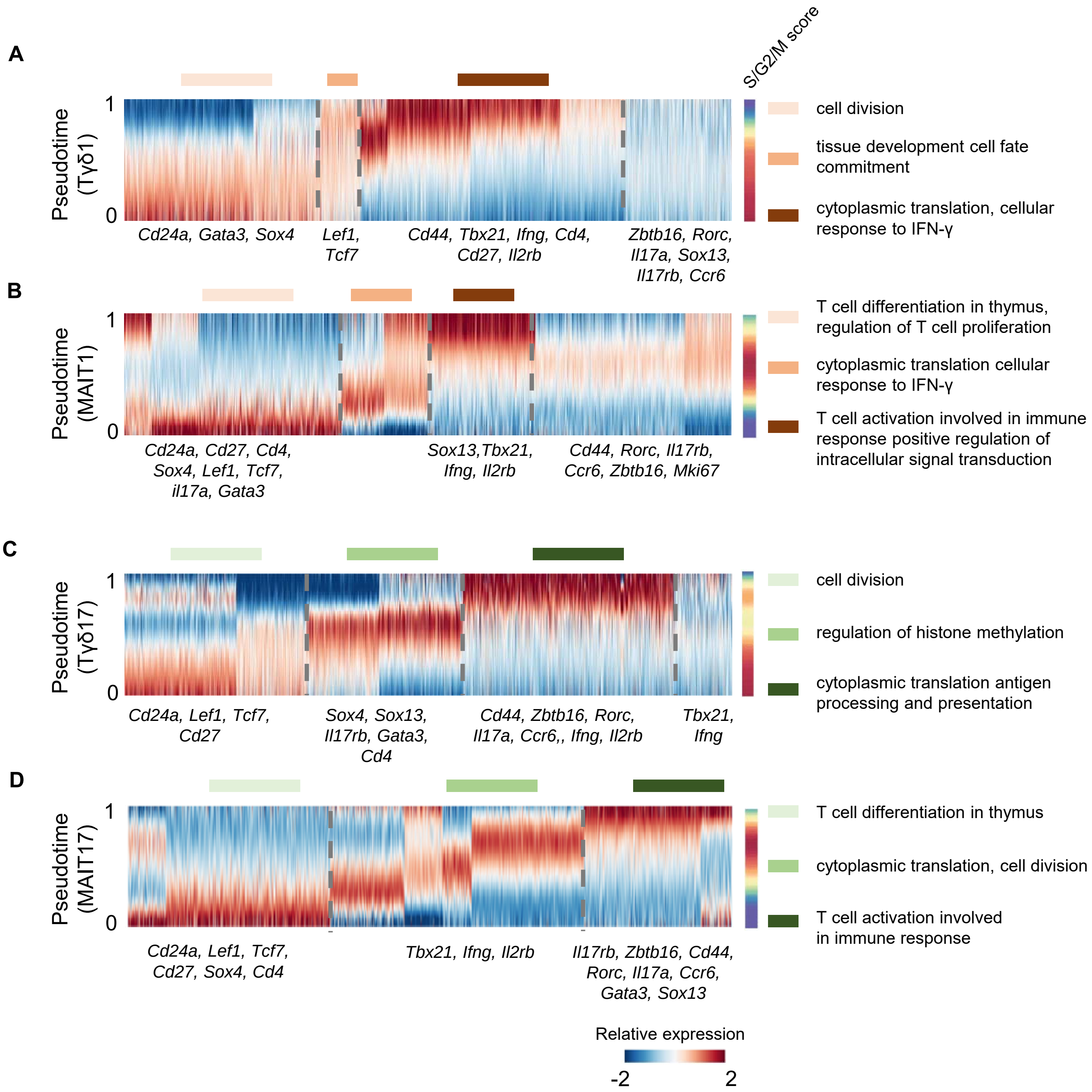

Supplementary Figure 17. Single-cell trajectory analysis of MAIT and γδ T cells. (A-D) Heat map illustrates gene expression trends of a union of marker genes of each cluster and subtype along differentiation trajectory towards Tγδ1 (A), MAIT1 (B), Tγδ17 (C) and MAIT17 (D). The enriched GO terms for each gene cluster separated by dashed gray lines are listed in the right panel. The S/G2/M cell cycle scores along the pseudotime trajectory, defined by the Z-score of the mean expression level of S/G2/M cell cycle related genes (Supplementary table 2), are shown in the graph.

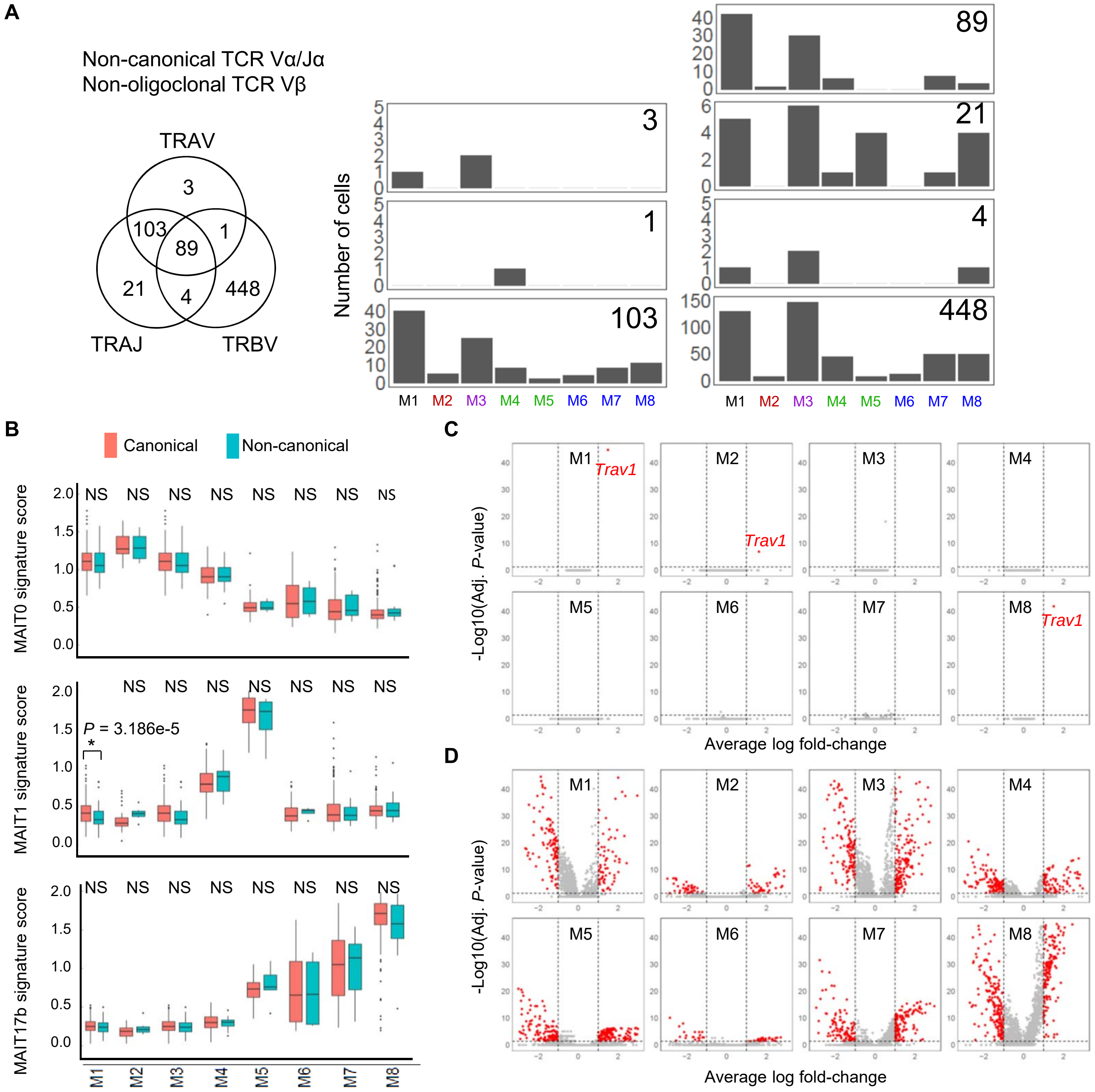

Supplementary Figure 18. Comparison between canonical and non-canonical MAIT cells. (A) Total 669 non-canonical and/or non-oligoclonal MAIT TCRs were analyzed and Venn-diagram shows numbers of cells with indicated TCRs. Graphs show the distribution of non-canonical and/or non-oligoclonal cells in each cluster, and each region of Venn-diagram can be matched with same cell numbers in the graphs. (B) Box plots show expression of MAIT0, MAIT1 and MAIT17b signature genes obtained from Legoux et. al. (Ref #13) in 1892 MAIT cells examined over 2 replicates with canonical and non-canonical TCRs in each cluster. *P<0.01; NS, non-significant (*P* > 0.01, two-sided Wilcoxon rank sum test). Inside the boxplot, the black line represents the median value and the size of the box is determined by the 25th and 75th percentiles of the data. The length of the whiskers is 1.5 times of IQR(Interquartile range). (C) Volcano plots showing differential gene expression profile between MAITs with canonical and non-canonical TCRs for each cell cluster of MAITs. (D) Volcano plots show differential gene expression profiles between MAITs with canonical TCRs and randomly selected CD4+/CD8+ DP cells (as a potential source of contamination of cells) in each cell cluster of MAITs. Differentially expressed genes (adjusted P-value < 0.05 ,log fold-change > 1 , two-sided Wilcoxon rank sum test) are highlighted in red (C-D).

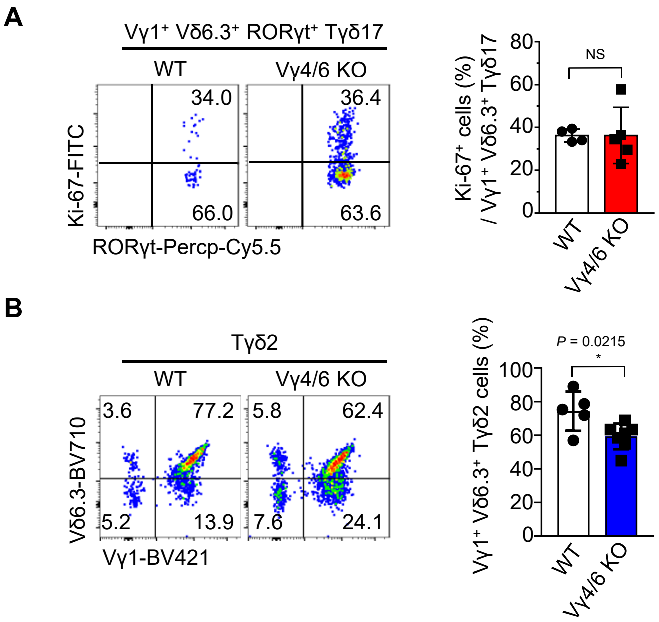

Supplementary Figure 19. Tγδ17 cells in WT and Vγ4/6 deficient mice. (A) Representative dot plots show Ki-67 expressions on CD24^{low} Vγ1^{+} Vδ6.3^{+} RORγt^{+} Tγδ17 cells from WT (left, n=4) or Vγ4/6 KO (right, n=5) mice and graph shows statistical analysis. Results are pooled from four independent experiments. (B) Representative dot plots show frequencies of Vγ1^{+} Vδ6.3^{+} cells among CD24^{low} PLZF^{high} Tγδ2 cells from WT (left, n=5) or Vγ4/6 KO (right, n=7) and graph shows statistical analysis. Representative results pooled from four independents. Numbers indicate frequencies of cells in adjacent gates. Data are presented as mean values ± SD. Unpaired two-tailed *t*-tests were used for data analysis. **P*<0.05. NS, not significant. Source data are provided as a Source Data file.

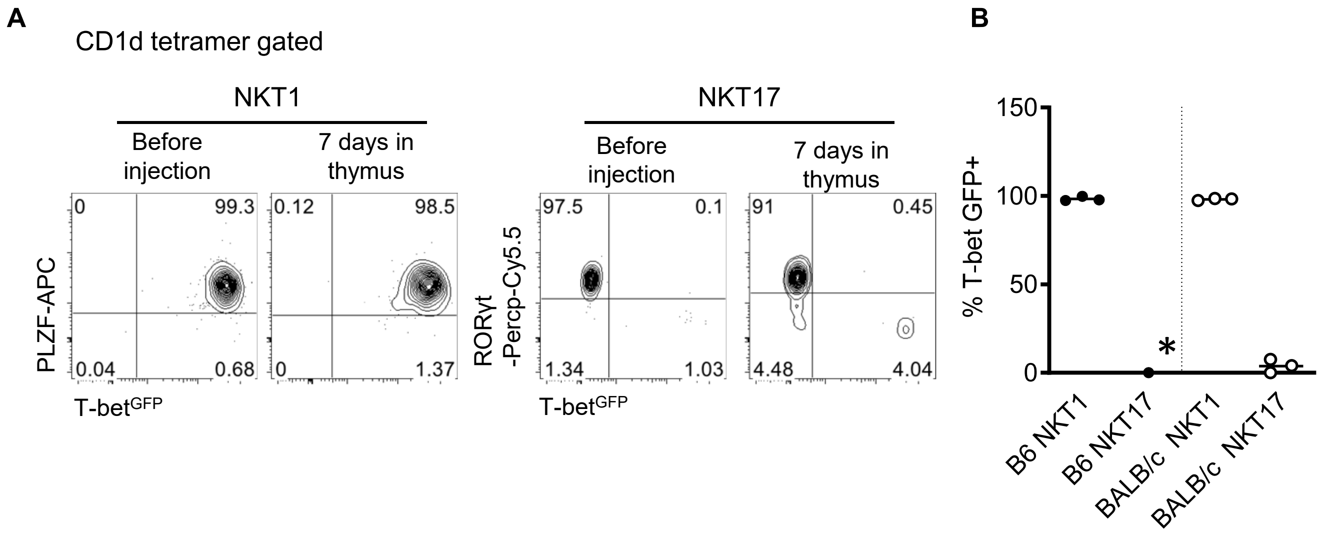

Supplementary Figure 20. NKT1 and NKT17 cells are terminally differentiated population. (A) Total thymocytes from either from B6 or BALB/c mice were enriched with CD1d tetramer and sorted as NKT1 (Tbx21^{gfp+}) and NKT17 (Tbx21^{gfp-} CD4^{-}) cells and stained with transcription factors before intrathymic injections. Sorted live cells were injected into congenic host and analyzed after 7 days. Representative plots show the phenotype of cells before and after injection of each population. Representative data of 3 independent experiments are shown. (B) Graph shows statistical analysis of GFP+ cells in donor population (N=3). *, two mice did not contain donor NKT cells. Source data are provided as a Source Data file.

		Total cells	TCR analysis
NKT	N1	225	202
	N2	210	188
	N3	843	769
	N4	785	657
	N5	871	690
	N6	68	22
	N7	283	247
	Total	3285	2775
MAIT	M1	532	440
	M2	83	48
	M3	562	494
	M4	154	134
	M5	148	137
	M6	113	106
	M7	231	209
	M8	464	324
	Total	2287	1892
γδ T	G1	308	201
	G2	334	218
	G3	295	184
	G4	741	461
	G5	267	143
	G6-1	155	101
	G6-2	74	68
	G7-1	417	232
	G7-2	76	57
	Total	2667	1665
Total		8239	6332

Supplementary Table 1. Number of cells analyzed.

S phase genes	G2/M phase genes
Dnajc2	*Dnajc2*
Mcm2	*Chek1*
Mcm3	*Ppm1d*
Mcm4	*Brca2*
Mki67	*Ccna1*
Mre11a	*Ccnb1*
Msh2	*Cdc25a*
Pcna	*Cdc25b*
Rad17	*Cdk2*
Rad51	*Nek2*
Sumo1	*Npm2*
	Pes1
	Prm1
	Rad21
	Ran
	Shc1
	Smc1a
	Stag1
	Terf1
	Psmg2
	Wee1

Supplementary Table 2. List of cell cycle related genes.
