# Supplementary material for: Single-cell RNA sequencing identifies shared differentiation paths of mouse thymic innate T cells
Source: Nat Commun. 2020 Aug 31;11:4367. doi: 10.1038/s41467-020-18155-8 (PMC7459300; doi:10.1038/s41467-020-18155-8)
Supplement: Supplementary file 3 — Reporting Summary [file 41467_2020_18155_MOESM3_ESM.pdf]

natureresearch

Corresponding author(s): Sanguk Kim, Jong Kyoung Kim, You Jeong Lee

Last updated by author(s): Aug 5, 2020

Reporting Summary

Nature Research wishes to improve the reproducibility of the work that we publish. This form provides structure for consistency and transparency in reporting. For further information on Nature Research policies, see Authors & Referees and the Editorial Policy Checklist .

Statistics

For all statistical analyses, confirm that the following items are present in the figure legend, table legend, main text, or Methods section.

n/a	Confirmed	
☐	☒	The exact sample size (n) for each experimental group/condition, given as a discrete number and unit of measurement
☐	☒	A statement on whether measurements were taken from distinct samples or whether the same sample was measured repeatedly
☐	☒	The statistical test(s) used AND whether they are one- or two-sided<br>*Only common tests should be described solely by name; describe more complex techniques in the Methods section.*
☒	☐	A description of all covariates tested
☒	☐	A description of any assumptions or corrections, such as tests of normality and adjustment for multiple comparisons
☐	☒	A full description of the statistical parameters including central tendency (e.g. means) or other basic estimates (e.g. regression coefficient) AND variation (e.g. standard deviation) or associated estimates of uncertainty (e.g. confidence intervals)
☐	☒	For null hypothesis testing, the test statistic (e.g. F, t, r) with confidence intervals, effect sizes, degrees of freedom and P value noted<br>*Give P values as exact values whenever suitable.*
☒	☐	For Bayesian analysis, information on the choice of priors and Markov chain Monte Carlo settings
☒	☐	For hierarchical and complex designs, identification of the appropriate level for tests and full reporting of outcomes
☒	☐	Estimates of effect sizes (e.g. Cohen's d, Pearson's r), indicating how they were calculated

Our web collection on statistics for biologists contains articles on many of the points above.

Software and code

Policy information about availability of computer code

Data collection	Single-cell RNA-sequencing data collection:<br>CellRanger v2.2.0, Dplyr v0.8.3, DropletUtils v1.2.2, Monocle 3 v0.2.0 R package, Org.Mm.eg.db v3.7.0, Palantir v0.2, Pheatmap v1.0.12, R v3.5.2, RColorBrewer v1.1-2, Reshape2 v1.4.3, Rstudio V1.2.1335, SC3 v1.10.1, Scater v1.10.1, Scmap v.1.4.1, Scran v1.10.2, Seurat v2.3.4, TopGO v2.34.0, Weblogo v3.7.1<br><br>Bulk RNA-sequencing data collection:<br>DESeq2 v1.22.1, EnhancedVolcano v1.0.0 R package, Ingenuity Pathway Analysis v2019, RSEM v1.3.1, RStudio v1.1.463, STAR v2.6.1-d
Data analysis	Flowjo v9/v10, GraphPad Prism v7/v8

For manuscripts utilizing custom algorithms or software that are central to the research but not yet described in published literature, software must be made available to editors/reviewers. We strongly encourage code deposition in a community repository (e.g. GitHub). See the Nature Research guidelines for submitting code & software for further information.

Data

Policy information about availability of data

All manuscripts must include a data availability statement. This statement should provide the following information, where applicable:

- Accession codes, unique identifiers, or web links for publicly available datasets
- A list of figures that have associated raw data
- A description of any restrictions on data availability

The authors declare that all data that support the findings of this study are included in the supplemental information and available upon reasonable requests to the corresponding authors. Sequencing data are available at the National Center for Biotechnology Information under accession number PRJNA549112 (Bulk RNA sequencing) and PRJNA549112 (Single-cell RNA sequencing)(https://www.ncbi.nlm.nih.gov/bioproject/?term=PRJNA549112) . To analyze iNKT, Th and ILC subsets, raw data of RNA-seq reads was downloaded from (SRA Project accession number: PRJNA318017 for iNKT, ArrayExpress accession number: E-MTAB-2582 for Th, and

GEO accession number: GSE85154 for ILC) and reanalyzed with the corresponded pipeline.

Field-specific reporting

Please select the one below that is the best fit for your research. If you are not sure, read the appropriate sections before making your selection.

☒ Life sciences ☐ Behavioural & social sciences ☐ Ecological, evolutionary & environmental sciences

For a reference copy of the document with all sections, see nature.com/documents/nr-reporting-summary-flat.pdf

Life sciences study design

All studies must disclose on these points even when the disclosure is negative.

Sample size	Sample size was not predetermined and we typically used 3-4 mice for each experiment with 2-3 times replication. We found that this setting is sufficient enough to identify significant difference between groups.
Data exclusions	No data were excluded.
Replication	All experiments were performed at least twice and similar results obtained. All attempts for replication were successful. Details are shown in the figure legends.
Randomization	For ex vivo experiments, we matched all age and sex for each experimental group. Animals were allocated for experiments randomly. For single-cell RNAseq experiment, we separated sex of mice to discriminate cell types.
Blinding	Blinding was not relevant to this study and investigators were not blinded to mouse genotypes prior to data acquisition.

Reporting for specific materials, systems and methods

We require information from authors about some types of materials, experimental systems and methods used in many studies. Here, indicate whether each material, system or method listed is relevant to your study. If you are not sure if a list item applies to your research, read the appropriate section before selecting a response.

Materials & experimental systems

n/a	Involved in the study	
☐	☒	Antibodies
☒	☐	Eukaryotic cell lines
☒	☐	Palaeontology
☐	☒	Animals and other organisms
☐	☒	Human research participants
☒	☐	Clinical data

Methods

n/a	Involved in the study	
☒	☐	ChIP-seq
☐	☒	Flow cytometry
☒	☐	MRI-based neuroimaging

Antibodies

Antibodies used

Mouse antibodies:
Anti-CD3ε APC-Cy7 (TONBO, clone 145-2C11, #25-0031-U100)
Anti-CD4 BUV395 (BD, clone GK1.5, #563790)
Anti-CD4 BV510 (BD, clone RM4-5, #563106)
Anti-CD8α BV650 (BD, clone 53-6.7, #563234)
Anti-CD19 PE-Cy7 (BD, clone 1D3, #552854)
Anti-CD24 BV605 (Biolegend, clone M1/69, #101827)
Anti-CD24 FITC (BD, clone M1/69, #553261)
Anti-CD24 PE/Cy7 (Biolegend, clone M1/69, #101821)
Anti-CD25 APC (Biolegend, clone PC61, #102012)
Anti-CD27 PerCP-eFluor710 (Thermo Fisher Scientific, clone LG.GF9, #46-0271-82)
Anti-CD44 PE (Biolegend, clone IM7, #103008)
Anti-CD44 redFluor710 (TONBO, clone IM7, #80-0441-U100)
Anti-CD45R/B220 BV711 (BD, clone RA3-6B2, #563892)
Anti-CD122 FITC (BD, clone TM-BETA 1, #553361)
Anti-CD122 PE (Thermo Fisher Scientific, clone TM-b1 (TM-beta1), #12-1222-81)
Anti-CD183 (CXCR3) PE-Cy7 (Biolegend, clone CXCR3-173, #126516)
Anti-CD186 (CXCR6) BV421 (Biolegend, clone SA051D1, #151109)
Anti-CD196 (CCR6) BV421 (Biolegend, clone 29-2L17, #129828)

Anti-CD279 (PD-1) APC (BD, clone J43, #562671)
Anti-IL-25R (IL-17RB) Alexa Fluor647 (Biolegend, clone 9B10, #146304)
Anti-IL-25R (IL-17RB) PE (Thermo Fisher Scientific, clone MUNC33, #12-7361-80)
Anti-γδ T-Cell Receptor BV421 (BD, clone GL3, #562892)
Anti-γδ T-Cell Receptor PE (BD, clone GL3, #553178)
Anti-γδ T-Cell Receptor PE-CF594 (BD, clone GL3, #563532)
Anti-Vγ1.1 (Heilig and Tonegawa's system: Vγ1) TCR BV421 (BD, clone 2.11, #566308)
Anti-Vγ1.1 + Vγ1.2 (Heilig and Tonegawa's system: Vγ1 + Vγ2) TCR PE (Biolegend, clone 4B2.9, #142704)
Anti-Vγ2 (Heilig and Tonegawa's system: Vγ4) TCR BV786 (BD, clone UC3-10A6, #742313)
Anti-Vγ3 (Heilig and Tonegawa's system: Vγ5) TCR BV510 (BD, clone 536, #743239)
Anti-Vγ5/Vδ1+ and Vγ6/Vδ1+ (Heilig and Tonegawa's system: Vγ5Vδ1+ and Vγ6Vδ1+) TCR rat IgM antibody (kindly provided by Dr. Robert Tigelaar, Yale University, 17D1)
Anti- Vγ7 TCR Biotinylated (kindly provided by Dr. Pablo Pereira, Institut Pasteur, clone F2.67)
Anti-TCRβ chain (BD, clone H57-597, #560656)
Anti-Vδ 6.3/2 TCR BV711 (BD, clone 8F4H7B7, #744476)
Purified CD16/32 (Biolegend, clone 93, #101302)
Anti-EOMES eFluor 450 (Thermo Fisher Scientific, clone Dan11mag, #48-4875-82)
Anti-IFN-γ PE-CF594 (BD, clone XMG1.2, #562303)
Anti-IL-4-Alexa647 (Biolegend, clone 11B11, #504110)
Anti-IL-4 BV421 (Biolegend, clone 11B11, #504119)
Anti-Ki-67 FITC (Thermo Fisher Scientific, clone SolA15, #11-5698-82)
Anti-IL-17A BV650 (BD, clone TC11-18H10, #564170)
Anti-PLZF Alexa Fluor647 (BD, clone R17-809, #563490)
Anti-PLZF PE-CF594 (BD, clone R17-809, #565738)
Anti-RORγt PerCP-Cy5.5 (BD, clone Q31-378, #562683)
Anti-RORγt PE-CF594 (BD, clone Q31-378, #562684)
Anti-T-bet PE-Cy7 (Thermo Fisher Scientific, clone eBio4B10, #25-5825-82)

Human antibodies
Anti-Human CD2 FITC (BD, clone RPA-2.10, #555326)
Anti-CD3 BUV395 (BD, clone UCHT1, #563546)
Anti-CD3 Alexa Fluor594 (Biolegend, clone UCHT1, #300446)
Anti-CD4 APC/Cy7 (Biolegend, clone OKT4, #317418)
Anti-CD8α Alexa Fluor700 (Biolegend, clone RPA-T8, #301028)
Anti-CD19 V500 (BD, clone HIB19, #561121)
Anti-CD45RA BV650 (BD, clone HI100, #563963)
Anti-CD45RO BV711 (BD, clone UCHL1, #563722)
Anti-CD161 Alexa Fluor488 (Biolegend, clone HP-3G10, #339924)
Anti-TCR Vα7.2 BV785 (Biolegend, clone 3C10, #351722)
Anti-TCR γ/δ PerCP/Cy5.5 (Biolegend, clone B1, #331224)
Anti-TCR Vγ9 BV421 (BD, clone B3, #744034)
Anti-TCR δ (TCR Vδ1-Jδ2) FITC (Thermo Fisher Scientific, clone TS-1, #TCR2055)
Anti-TCR Vδ2 FITC (Biolegend, clone B6, #331406)
Anti-IFN-γ BUV395 (BD, clone B27, #563563)
Anti-IL-4 BV605 (Biolegend, clone MP4-25D2, #500827)
Anti-IL-17A APC (Thermo Fisher Scientific, clone eBio64DEC17, #17-7179-42)
Anti-T-bet PE-Cy7 (Thermo Fisher Scientific, clone eBio4B10, #25-5825-82)
Anti-PLZF Alexa Fluor647 (BD, clone R17-809, #563490)
Anti-RORγt PE (BD, clone Q21-559, #563081)

Secondary antibodies
Anti-rat IgM FITC (Biolegend, clone MRM-47, #408905)
Streptavidin APC-Cy7 (BD Pharmingen, #554063)
Streptavidin PE (BD Pharmingen, #554061)
Streptavidin-R-Phycoerythrin (ProZyme, PJRS25)

Other
Anti-PE MicroBeads Ultrapure (Miltenyi Biotec, #130-105-639)

Validation

Most of the antibodies used are commercially available and have been routinely tested by manufacturers. All antibodies were tested in the laboratory using on known positive and negative controls and titrated prior to all experiments. Anti-Vγ5Vδ1+ and Vγ6Vδ1+ TCR rat IgM antibody (kindly provided by Dr. Robert Tigelaar, Yale University, 17D1) and Anti- Vγ7 TCR Biotinylated (kindly provided by Dr. Pablo Pereira, Institut Pasteur, clone F2.67) antibody were also tested using positive and negative controls to determine the specificity of those antibodies. FMO (Fluorescence Minus One) or a particular isotype control staining group was used to determine the staining positivity.

Animals and other organisms

Policy information about studies involving animals; ARRIVE guidelines recommended for reporting animal research

Laboratory animals	The following mice were used and maintained on a C57BL/6 bakcground : B6 (C57BL/6J; Tcrd−/− B6; Tbx21−/− B6 ; B6 Rorc-Cre; B6 Il17a-Cre; B6.Cg-Gt(ROSA)26Sortm14(CAG-tdTomato)Hze/J; B6.Cg-Gt(ROSA)26Sortm6(CAG-ZsGreen1)Hze/J; Vγ4/6-/-; Cd4Cre Gata3 f/f mice<br>The following mice were used and maintained on a BALB/c bakcground : BALB/cJ; BALB/cByJ; KN2/KN2; BALB/c Rorc(γt)-EGFP<br>Thymic cell populations were isolated from 6-12 week-old mice with exception of newborn mice (7 day-old) used in Figure 5D. KN2 and Tbx21gfp reporter mice were previously described (ref# 8) and B6.Cg-Gt(ROSA)26Sortm6(CAG-ZsGreen1)Hze/J mice were received from Dr. Charles D. Surh (POSTECH, Korea). Vγ4/6 KO mice were kindly provided by Dr. Rebecca O'Brien (National Jewish Health, USA) under the permission from Koichi Ikuta (Kyoto University, Japan). All mice were used at the age of 6-12 weeks unless indicated and age- and sex-matched animals were used as controls. In experiments analyzing Tbx21−/− B6 or Cd4Cre Gata3f/f mice, littermate controls were bred in same cages. In experiments analyzing Tcrd-/- B6, Vγ4/6 KO, or BALB/c Rorc(γt)EGFP mice, WT control mice were bred separately. Both female and male mice were used in experiments. In oder to isolate intact thymi, experimental mice were euthanized by carbon dioxide. All animals were bred and maintained in a specific pathogen-free (SPF) conditions, ambient temperature 23±1°C, humidity 50±10% and a dark/ light cycle of 12 hours.
Wild animals	No wild animals were used in this study.
Field-collected samples	No field collected samples were used in this study.
Ethics oversight	All in vivo mouse experiments were approved by the Institutional Animal Cared and Use Committees (IACUC) of the Pohang University of Science and Technology (POSTECH).

Note that full information on the approval of the study protocol must also be provided in the manuscript.

Human research participants

Policy information about studies involving human research participants

Population characteristics	Human liver perfusates was obtained from healthy living liver transplant donors who were hepatitis B virus (HBV) DNA, hepatitis C virus (HCV), and anti-human immunodeficiency virus (HIV) antibody negative. Healthy individuals had a median age of 39 years (range 20-59), including both females and males.
Recruitment	Participants were recruited among healthy donors of liver transplantation. Each participant provided informed consent according to Helsinki Declaration, and all uses of human material have been approved by the institutional review board of Severance Hospital (Seoul, Republic of Korea; 2013-1071-001). The human liver perfusates were selected with simple random sampling.
Ethics oversight	The study was approved by the institutional review board of Severance Hospital (Seoul, Republic of Korea; 2013-1071-001) and conducted according to the principles of the Declaration of Helsinki.

Note that full information on the approval of the study protocol must also be provided in the manuscript.

Flow Cytometry

Plots

Confirm that:

- [x] The axis labels state the marker and fluorochrome used (e.g. CD4-FITC).
- [x] The axis scales are clearly visible. Include numbers along axes only for bottom left plot of group (a 'group' is an analysis of identical markers).
- [x] All plots are contour plots with outliers or pseudocolor plots.
- [x] A numerical value for number of cells or percentage (with statistics) is provided.

Methodology

Sample preparation	Mouse thymus cells were harvested using mechanical dissociation through a 70㎛ cell strainer, washed and resuspended in RPMI medium plus 10% (vol/vol) FCS. The number of cells and viability were determined by an automated cell counting (Beckman Coulter Vi-CELL). Zombie Aqua Fixable Viability Kit (BioLegend, #423101) was used to exclude dead cells. Afterwards, cells were incubated with fluorochrome-conjugated antibody cocktail for surface markers on ice (or at 4℃) for 30 min. Cells were subsequently washed with FACS buffer (DPBS+2% FCS). For staining of nuclear transcription factors and cytokines after stimulation, we used eBioscience kit (#00-5523-00) and followed by manufacturer's instructions.<br><br>Of the 1000 ml of total human liver perfusate, the first 500 ml was discarded and the second 500 ml collected and filtered. Liver sinusoidal mononuclear cells (LSMCs) were isolated by density gradient centrifugation using Ficoll-Paque (GE Healthcare Life Science). Human LSMCs was blocked with 1% Normal mouse serum (stock: 50㎎/㎖) and followed by surface antigen staining. After subsequent washing, fixed human liver cells were incubated with antibodies for intracellular target markers.

Instrument	LSR II (Becton Dickinson) for analysis and MoFlo Astrios (Beckman Coulter) for sorting
Software	Flowjo software v9 and v10 (Treestar) were used to collect and analyze data.
Cell population abundance	For bulk and single cell RNAseq, we used CD1d and MR1 tetramer positive cells and GL3+ gamma delta T cell subsets with 95-99% purity as determined by flow cytometry in supplementary figure 2.
Gating strategy	For all experiments, we identified cells by gating on lymphocytes using FSC-A and SSC-A. Doublets were excluded using SSC-A and SSC-W. B220+ cells were gated out and MR1 and CD1d tetramer or GL3+ gamma delta positive cells were used for cell sorting.

☒ Tick this box to confirm that a figure exemplifying the gating strategy is provided in the Supplementary Information.
