# Supplementary material for: Single-cell RNA sequencing identifies shared differentiation paths of mouse thymic innate T cells
Source: Nat Commun. 2020 Aug 31;11:4367. doi: 10.1038/s41467-020-18155-8 (PMC7459300; doi:10.1038/s41467-020-18155-8)
Supplement: Supplementary file 4 — Description of Additional Supplementary Files [file 41467_2020_18155_MOESM4_ESM.pdf]

Description of Additional Supplementary Files

Supplementary Data 1. List of subset specific signature genes
Supplementary Data 2. List of TCR clonotypes
